# Associations between physical frailty and living arrangements in Japanese older adults living in a rural remote island: The Shimane CoHRE study

**DOI:** 10.1002/jgf2.544

**Published:** 2022-04-10

**Authors:** Ryo Miyazaki, Takafumi Abe, Shozo Yano, Kenta Okuyama, Naoki Sakane, Hitoshi Ando, Minoru Isomura, Masayuki Yamasaki, Toru Nabika

**Affiliations:** ^1^ Faculty of Human Sciences Shimane University Matsue‐shi Japan; ^2^ Center for Community‐Based Healthcare Research and Education (CoHRE), Organization for Research and Academic Information Shimane University Izumo‐shi Japan; ^3^ Center for Primary Health Care Research Lund University Malmö Sweden; ^4^ Division of Preventive Medicine, Clinical Research Institute for Endocrine and Metabolic Disease, Kyoto Medical Center National Hospital Organization Kyoto Japan; ^5^ Department of Cellular and Molecular Function Analysis Kanazawa University Graduate School of Medical Sciences Kanazawa Japan

**Keywords:** elderly, gait speed, Japan, living alone, marital status

## Abstract

**Background:**

Living arrangements have been known to be associated with physical frailty. However, the prevalence of frailty and its risk factors in remote islands is not understood. We examined the association between living arrangements and objectively measured frailty among older adults living in a remote island of Japan.

**Methods:**

Among older people living in Okinoshima, 656 older adults (75.6 ± 6.4 years) were analyzed. Physical frailty (robust, prefrailty, or frailty) was assessed using the 5‐item frailty phenotype (unintentional weight loss, self‐reported exhaustion, weakness, slow walking speed, and low physical activity). Physical functions (muscle mass, gait speed, and grip strength) were measured objectively.

**Results:**

The prevalence of frailty and prefrailty was 6.6% and 43.8%, respectively. Living with a spouse resulted in a significantly lower prevalence of frailty (*p* < 0.001) compared with other living arrangements. All objectively measured physical functions among those who lived with a spouse were significantly superior to those who lived with family or alone (*p* < 0.001). Multinomial logistic regression showed that living alone was significantly associated with frailty (odds ratio [OR] 2.36, 95% confidence interval [CI] 1.07–5.24) and prefrailty (OR 1.75, 95% CI 1.14–2.69) after adjusting for all covariates.

**Conclusion:**

The prevalence of frailty on remote islands seemed similar to that in urban areas. Older people living in remote islands might be able to maintain their physical health. Furthermore, living alone may correlate with increased risks of frailty and prefrailty. Among elderly individuals on remote islands, living with a spouse might be desirable to prevent (pre)frailty.

## Graphical abstract

The prevalence of physical frailty and its risk factors in remote islands is not understood. We observed that the prevalence of frailty in remote islands seemed similar to that of urban areas. Moreover, we found that living alone may possess higher risks for frailty and prefrailty in such islands.

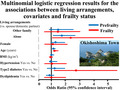

## 1 INTRODUCTION

Japanese land is a unique island country, featuring 6852 islands, which is the 7th largest number of islands in the world. Among these Japanese islands, 416 are considered as remote islands and approximately 600,000 people live in these remote islands.^1^ Previous studies have reported that residents living in remote islands or rural areas have been experiencing challenges regarding access to medical services including nurses, as well as poorer health outcomes compared with urban residents.^2^ Moriyama et al.^3^ reported that remote islands in Japan have been reported to face challenges because of the shortage of primary care practitioners, specialist physicians, and nurses. Although such health disadvantages in these remote islands are prominent in Japan, evidence regarding “remote health” is quite limited.

Moreover, the living arrangements, especially living alone, on remote islands may be critical for older adults to maintain their health. It is estimated that by 2040, 17.7% of Japanese households will comprise older adults living alone, an increase from 11.7% in 2015.^4^ Living alone is known to be strongly associated with serious health impairments (e.g., elevated risk of dementia^5^ and weak cognitive functions).^6^ While urban areas are often well situated to accommodate individuals living alone, with greater availability of small apartments and resources catering to single people, living alone may have more adverse effect on health in rural areas, where there are fewer resources and housing units to appropriately and affordably accommodate such households, especially on remote islands. Although the size of the aging population is estimated to increase further in several countries, with the rate particularly high in rural settings, such as remote islands,^7^ studies targeting such settings are very limited at present. Based on these backgrounds, investigating health and living arrangements in rural remote islands would be significant to discuss health policies to improve rural remote health.

Physical frailty (frailty) is a geriatric syndrome with multiple causes and contributors.^8^ Fried et al.^8^ defined frailty as a predominantly physical condition requiring the presence of 3 or more of the following 5 components: weight loss, exhaustion, weakness, slowness, and low physical activity. Frailty increases hospitalization risk^9^ and eventually mortality risk.^10^ Globally, using the Fried frailty phenotype, frailty prevalence has been reported to be 12% in 22 European countries,^11^ 19.6% in Latin America and the Caribbean,^12^ and 8% in China.^13^ In Japan, 7.4% of community‐dwelling older adults have frailty.^14^ As the aging rate of the population in Japan is the highest in the world and continues to increase, there will be an increasing number of frail older adults. However, almost no data were available on remote islands. Only one study has reported that the prevalence of frailty was 16.1% on remote islands in Japan.^15^ Because this number was computed using questionnaires, the prevalence of frailty using internationally validated methods on remote islands in Japan is unknown.

Living alone has been reported to be associated with frailty. Recent systematic reviews identified both living alone and marital status as an important risk factor for physical frailty.^16^, ^17^ In their review, frailty risk among older people living alone was 28% higher than those living with someone.^16^ Regarding marital status, the article above showed that unmarried individuals were almost twice more likely to be frail than married individuals.^17^ This evidence suggests that both living alone and marital status should be recognized as important factors of frailty. Nevertheless, such association has not been investigated in rural remote islands where a greater effect of living alone could be hypothesized. Taken together, the associations between frailty status and living alone in remote island with validated measurements would become important data for remote health policies.

Based on these backgrounds, the present study investigated associations between physical frailty and differences in living arrangements among older adults who live in a remote island in Shimane, Japan.

## 2 METHODS

### 2.1 Design

The data for this cross‐sectional study were collected as a part of the Shimane CoHRE study in June 2018. The Shimane CoHRE study, which aimed to examine the determinants of lifestyle‐related diseases such as hypertension, diabetes, cardiovascular diseases,^18^ and depression,^19^ was conducted by Shimane University in collaboration with a health examination program in Okinoshima Town, Japan. Health examination programs are conducted annually for individuals aged 40 years and older who are covered by National Health Insurance. The study protocol was approved by the Ethics Committee of Shimane University School of Medicine in 2017 (number 3193). Written informed consent was obtained from all participants. In all, 699 older adults aged 65 years or older participated in the health examinations. Participants with missing data (*n* = 43) were excluded; consequently, data from 656 participants were analyzed.

### 2.2 The description of Okinoshima Town

Okinoshima Town is a remote island in the Japanese Sea and is one of the Oki Islands in Shimane Prefecture, Japan, and has been designated under the Remote Islands Development Act. The island is located 44 km from the main island of Shimane (150 min by ship). In 2017 when the research was commenced, the population was 14,374 (13,768 in 2021), and 39.6% (41.0% in 2021) of them were 65 years or older.^20^ The population has decreased by about 49% since 1976. There were one general hospital and 14 primary care clinics.

### 2.3 Living arrangements

Trained fieldworkers conducted structured interviews and recorded living arrangements. Living arrangements were ascertained from the self‐reported question “What best describes your current situation?” according to the previous study.^21^ Response options were single, married/living with partner, widowed, divorced, and living with other families except spouse (children and grandchildren). Participants were classified into three groups: living with a spouse/partner (married/living with a partner) and living with families but not living with a spouse/partner (including single, widowed, divorced, separated), or living alone. Living alone was defined as being the only person in the household, independent of marital status, number of children, and number of friends and/or relatives.^21^, ^22^

### 2.4 Physical frailty

Frailty was determined using the Japanese version of the Cardiovascular Health Study (J‐CHS).^23^ Because of its convenience, this criterion has been used in community‐based settings in many previous studies.^24^, ^25^ We considered the physical frailty phenotype to be characterized by the presence of three or more of the following five conditions: slowness, weakness, exhaustion, low activity levels, and unintentional weight loss. Participants not reporting any of these conditions were considered nonfrail (robust), while those with one or two were considered prefrailty. Slowness and weakness were determined by objectively measured physical functions (gait speed and grip strength). Grip strength was measured using a Smedley‐type handheld dynamometer (GRIPD; Takei, Niigata, Japan). Gait parameters and foot pressure patterns were measured using a sheet‐type pressure sensor (45.5 × 183 cm plate sensor; Healthwalk; Kao Corporation) placed in the center of a 5 m walkway. Participants were asked to walk freely along a 7 m course and return to the starting point. The average gait speed to complete the distance for both right and left feet was used for the analysis.^26^ This pressure sensor has been used in previous studies,^24^, ^27^ showing its convenience and association with various health parameters. Slow gait speed was identified based on a cutoff of <1.0 m/s. Weakness was identified using maximum grip strength, applying a gender‐specific cutoff (<26 kg for men and <18 kg for women). Exhaustion was identified by a “yes” response to the following question: “In the last two weeks, have you felt tired for no reason?” Physical activity was evaluated through the following questions: (1) “Do you engage in moderate levels of physical exercise or sports to improve your health?” and (2) “Do you engage in low levels of physical exercise to improve your health?” Participants who answered “no” to both questions were categorized into the low‐activity category. Weight loss was identified by a “yes” response to the question, “Have you lost 2 kg or more in the past six months?”.

### 2.5 Other parameters

Muscle mass was measured through bioelectrical impedance assay using the MC‐780A (Tanita, Tokyo, Japan). The whole‐body impedance was measured using an ipsilateral foot‐hand electrical pathway. Appendicular muscle mass was calculated as the sum of the muscle mass of the arms and legs. Absolute appendicular muscle mass was converted to skeletal muscle index (SMI) by dividing the value by the square of height in meters (kg/m^2^). Data on gender (men or women), age, and medication‐taking were obtained using a questionnaire of the health examination. Cardiovascular disease (CVD) risk markers were defined as follows, based on Japanese guidelines: hypertension (systolic blood pressure ≥ 140 mmHg and/or diastolic blood pressure ≥ 90 mmHg),^28^ type 2 diabetes (hemoglobin A1c ≥ 6.5%),^29^ and dyslipidemia (low‐density lipoprotein cholesterol ≥140 mg/dl, high‐density lipoprotein cholesterol <40 mg/dl, and/or triglyceride ≥150 mg/dl),^30^ or receiving medication for these diseases. From the obtained data, body mass index (BMI) was calculated by dividing the body weight by the squared height (kg/m^2^).

### 2.6 Statistical analysis

Basic demographic and health characteristics of study subjects were described by total and by gender. We have further described and compared the demographic and frailty‐related health characteristics by living arrangements. We used one‐way ANOVA and Bonferroni post hoc correction for quantitative variables, such as age, BMI, and muscle mass, and used a chi‐squared test for categorical variables, such as physical inactivity and exhaustion. Multinomial logistic regression models were used to calculate odds ratios (ORs) and 95% confidence intervals (CIs) for the association between living arrangements and frailty status. An unconditional logistic regression analysis was performed. Then, crude (simple) and adjusted (age, BMI, and CVD risk factors such as hypertension, diabetes, and dyslipidemia were included as covariates) models were employed. Furthermore, subgroup analyses were performed to clarify whether or not the relationship between living arrangements and frailty differed depending on gender. Statistical analyses were performed using SPSS version 23 (IBM Corp.). All *p*‐values for statistical tests were two‐tailed, and values <0.05 were regarded as indicating statistical significance.

## 3 RESULTS

### 3.1 Sample characteristics

The demographic and health characteristics of the study population are listed in Table 1. Overall, 656 participants (245 men and 411 women, mean age: 75.6 ± 6.4 years, mean BMI: 23.0 ± 3.1 kg/m^2^) participated in the community‐based health examination. Of these, 6.6% (men, 6.5%; women, 6.6%) had frailty and 43.8% (men, 38.8%; women, 46.7%) had prefrailty. A total of 131 (20.0%), 85 (13.0%), and 440 (67.0%) older adults were reported living alone, living with other family, and living with spouse/domestic partner, respectively.

**TABLE 1:** Demographic and health characteristics among participants

	All (*n* = 656)		Men (*n* = 245)		Women (*n* = 411)	
	Mean	SD	Mean	SD	Mean	SD
Age (years)	75.6	6.4	75.2	6.5	75.8	6.3
Gender (male %)	(245, 37.3%)					
Anthropometrics						
Body mass index (kg/m^2^)	23.0	3.1	23.3	2.9	22.9	3.2
Body fat (%)	26.4	8.5	20.1	6.4	30.2	7.2
Physical functions						
Muscle mass (kg)	16.2	3.9	20.2	2.9	13.8	2.1
Skeletal muscle index (kg/m^2^)	6.7	1.1	7.6	0.9	6.2	0.8
Grip strength (kg)	27.0	7.7	34.6	6.1	22.4	4.2
Gait speed (cm/s)	112.4	22.0	112.1	18.8	112.6	23.7
	***n***	**%**	***n***	**%**	***n***	**%**
Frailty status						
Robust	326	49.7	134	54.7	192	46.7
Prefrailty	287	43.8	95	38.8	192	46.7
Frailty	43	6.6	16	6.5	27	6.6
Medication						
Hypertension receiving medication	355	54.1	126	51.4	229	55.7
Type 2 diabetes receiving medication	65	9.9	35	14.3	30	7.3
Dyslipidemia receiving medication	196	29.9	42	17.1	154	37.5
With whom you live						
Alone	131	20.0	31	12.7	100	24.3
Other family	85	13.0	16	6.5	69	16.8
Spouse/domestic partner	440	67.0	198	80.8	242	58.9

*Note:* Data are mean ± standard deviation (SD). Basic demographic and health characteristics of study subjects were described by total and by gender.

### 3.2 Living arrangements and frailty‐associated parameters

Living arrangements were associated with a significantly higher prevalence of frailty (*p* < 0.001, Table 2). Post hoc test results showed that those who live alone had lower physical functions (skeletal muscle mass, gait speed, and grip strength) compared with the other groups (*p* < 0.05), while physical functions among those who live alone and those who live with other family did not differ (Table 2, Table S1).

**TABLE 2:** Comparison between body compositions, physical functions, and frailty parameters with living arrangements

	With whom you live	Spouse/domestic partner (*n* = 440)		Other family (*n* = 85)		Alone (*n* = 131)		*p*‐Value	Pairwise comparisons
		Mean	SD	Mean	SD	Mean	SD		
Age (years)		74.6	6.0	78.4	7.1	76.9	6.4	<0.001	*: SP vs. FA, *: SP vs. AL
Body mass index (kg/m^2^)		23.1	3.1	22.9	3.0	22.9	3.2	0.762	
Muscle mass (kg)		16.9	3.9	14.5	3.4	15.1	3.6	<0.001	*: SP vs. FA, *: SP vs. AL
Skeletal muscle index (kg/m^2^)		6.9	1.1	6.3	1.0	6.4	1.1	<0.001	*: SP vs. FA, *: SP vs. AL
Grip strength (kg)		28.4	7.9	24.1	6.2	24.0	6.8	<0.001	*: SP vs. FA, *: SP vs. AL
Gait speed (cm/s)		114.3	20.9	108.0	22.4	108.7	24.6	0.006	*: SP vs. FA, *: SP vs. AL
		***n***	**%**	***n***	**%**	***n***	**%**	***p*‐value**	
Frailty									
Robust (*n*, %)		245	55.7	33	38.8	48	36.6	<0.001	
Prefrailty (*n*, %)		173	39.3	45	52.9	69	52.7		
Frailty (*n*, %)		22	5.0	7	8.2	14	10.7		
Frailty Index items									
Weight loss		38	8.6	11	12.9	22	16.8	0.023	
Exhaustion		70	15.9	17	20.0	22	16.8	0.659	
Physical inactivity		49	11.1	12	14.1	15	11.5	0.733	
Lower grip strength		34	7.7	14	7.7	32	24.4	<0.001	
Lower gait speed		107	24.3	26	24.3	41	31.3	<0.001	

*Note:* Data are mean ± standard deviation (SD). We compared the prevalence by living arrangements using a one‐way analysis of variance for quantitative data and a chi‐squared test for categorical data.

Abbreviations: AL, alone; FA, other family; SP, spouse/domestic partner.

*p < 0.05.

Table 3 shows the results of multinomial logistic regression analysis. Model 1 (crude) showed that both living alone and living with other family were significantly associated with higher risk of prefrailty (*p* < 0.05) when compared to living with spouse/partner. In Model 1, living alone was significantly associated with higher risk of frailty (*p* < 0.05). Model 2 (adjusted) showed that living alone was significantly associated with not only frailty (odds ratio (OR) 2.36, 95% confidence interval (CI) 1.07–5.24) but also prefrailty (OR 1.75, 95% CI 1.14–2.69) after adjusting for covariates including age, BMI, and chronic diseases. In the adjusted model, the frailty risk among those who live with other family was not significant. Table 4 shows the association between living arrangements and frailty by gender. Among men, the adjusted model showed significant associations of living with other family (OR 10.17, 95% CI 1.56–66.26) and living alone (OR 12.32, 95% CI 2.94–51.67) with frailty. Among women, living alone was likely to be associated with prefrailty (OR 1.86, 95% CI 1.11–3.11).

**TABLE 3:** Multinomial logistic regression results for the associations between living arrangements, age, body mass index, chronic diseases, and frailty status

	Crude model		Adjusted model	
	Prefrailty	Frailty	Prefrailty	Frailty
	OR (95% CI)	OR (95% CI)	OR (95% CI)	OR (95% CI)
With whom you live (ref = Spouse/domestic partner)				
Spouse/domestic partner	1.00 (reference)	1.00 (reference)	1.00 (reference)	1.00 (reference)
Other family	**1.93 (1.18–3.15)**	2.36 (0.94–5.96)	1.51 (0.90–2.52)	1.45 (0.53–3.07)
Alone	**2.04 (1.34–3.09)**	**3.25 (1.55–6.80)**	**1.75 (1.14–2.69)**	**2.36 (1.07–5.24)**
Covariates				
Gender, women			1.33 (0.93–1.89)	1.07 (0.53–2.16)
Age (years)			**1.06 (1.03–1.09)**	**1.15 (1.09–1.21)**
Body mass index (kg/m^2^)			1.00 (0.95–1.05)	0.94 (0.84–1.05)
Hypertension or receiving associated medication			0.99 (0.70–1.42)	1.05 (0.52–2.08)
Type 2 diabetes or receiving associated medication			1.22 (0.75–1.98)	**2.53 (1.18–5.71)**
Dyslipidemia or receiving associated medication			0.86 (0.62–1.21)	1.04 (0.52–2.08)

*Note:* Living arrangements were analyzed using multinomial logistic regression. Bold shows significance, *p* < 0.05. Age and body mass index are continuous variables. Adjusted model: Gender, age, body mass index, and diagnosis of diseases (hypertension, type 2 diabetes, or dyslipidemia) and/or being under medications were adjusted. Hypertension (systolic blood pressure ≥ 140 mmHg and/or diastolic blood pressure ≥ 90 mmHg; the Japanese Society of Hypertension Guidelines for the Management of Hypertension). Type 2 diabetes (hemoglobin A1c ≥ 6.5%; Japanese Clinical Practice Guideline for Diabetes 2016).

Abbreviations: CI, confidence interval; OR, odds ratio.

**TABLE 4:** Gender‐stratified results of multinomial logistic regression results for the associations between the living arrangements, age, body mass index, chronic diseases, and frailty status

			Crude model		Adjusted model	
			Prefrailty	Frailty	Prefrailty	Frailty
			OR (95% CI)	OR (95% CI)	OR (95% CI)	OR (95% CI)
Men (*n* = 245)						
With whom you live (ref = spouse/domestic partner)						
Spouse/domestic partner			1.00 (reference)	1.00 (reference)	1.00 (reference)	1.00 (reference)
Other family			1.74 (0.56–5.38)	**9.58 (1.91–47.98)**	1.74 (0.56–5.45)	**10.17 (1.56–66.26)**
Alone			1.26 (0.54–5.38)	**10.32 (3.01–35.4)**	1.40 (0.58–3.38)	**12.32 (2.94–51.67)**
Covariates						
Gender, women						
Age (years)					1.03 (0.99–1.07)	**1.17 (1.05–1.29)**
Body mass index (kg/m^2^)					0.95 (0.86–1.05)	0.78 (0.62–0.98)
Hypertension or receiving associated medication					0.88 (0.49–1.57)	1.15 (0.27–4.80)
Type 2 diabetes or receiving associated medication					1.23 (0.61–2.45)	2.70 (0.75–9.66)
Dyslipidemia or receiving associated medication					0.99 (0.58–1.69)	2.01 (0.53–7.67)
Women (*m* = 411)						
With whom you live (ref = Spouse/domestic partner)						
Spouse/domestic partner			1.00 (reference)	1.00 (reference)	1.00 (reference)	1.00 (reference)
Other family			**1.91 (1.09–3.34**	1.20 (0.37–3.89)	1.41 (0.78–2.55)	0.62 (0.18–2.17)
Alone			**2.24 (1.37–3.68)**	1.63 (0.62–4.26)	**1.86 (1.11–3.11)**	1.03 (0.37–2.85)
Covariates						
Gender, women						
Age (years)					**1.07 (1.03–1.11)**	**1.17 (1.08–1.25)**
Body mass index (kg/m^2^)					1.02 (0.96–1.09)	1.02 (0.89–1.16)
Hypertension or receiving associated medication					1.12 (0.72–1.75)	0.89 (0.36–2.23)
Type 2 diabetes or receiving associated medication					1.19 (0.59–2.36)	2.34 (0.75–7.30)
Dyslipidemia or receiving associated medication					0.77 (0.49–1.19)	0.92 (0.38–2.22)

*Note:* Living arrangements were analyzed using multinomial logistic regression. Bold shows significance, *p* < 0.05. Age and body mass index are continuous variables. Adjusted model: Gender, age, body mass index, and diagnosis of diseases (hypertension, type 2 diabetes, or dyslipidemia) and/or being under medications were adjusted. Hypertension (systolic blood pressure ≥ 140 mmHg and/or diastolic blood pressure ≥ 90 mmHg; the Japanese Society of Hypertension Guidelines for the Management of Hypertension). Type 2 diabetes (hemoglobin A1c ≥ 6.5%; Japanese Clinical Practice Guideline for Diabetes 2016).

Abbreviations: CI, confidence interval; OR, odds ratio.

## 4 DISCUSSION

In the present study, we found that living alone was associated with physical frailty among older adults living in Okinoshima Town, a remote island in Japan. To the best of our knowledge, this is the first study investigating the prevalence of frailty and the associations between several forms of living arrangements and frailty (frailty and prefrailty) among older adults living in a rural remote island. We believe that our findings would be useful for policymaking to improve rural remote health.

In our sample, the prevalence of frailty and prefrailty was 6.6% and 43.8%. According to a systematic review and meta‐analysis, in Japan, 7.4% and 48.1% of community‐dwelling older adults have frailty and prefrailty, respectively.^14^ According to a recently published study comparing frailty prevalence between different locations in Japan among more than 9000 community‐dwelling older adults assessed using a questionnaire, the frailty prevalence in rural areas was 15.1%, while the numbers were 22.4% in metropolitan areas and 14.1% in suburban areas.^31^ Regarding island data, Yamanashi et al.^15^ reported the frailty prevalence in rural island in Nagasaki was 16.1%. However, all frailty data in the studies above were evaluated by questionnaires.^15^, ^31^ Based on our findings in which frailty was evaluated by both objective and subjective measures in alignment with the validated frailty index, the prevalence of frailty in remote island might be similar to that of urban areas, even though remote islands have higher older population rates and poorer access to healthcare services. The unique lifestyle (such as dietary and/or exercise habits) among residents of remote islands might be the reason for the findings of the present study. Regarding the dietary lifestyle, a study conducted in a rural coastal district of Japan found that fish consumption was associated with a reduced risk of coronary heart diseases.^32^ Furthermore, the prevalence of physical inactivity among those who live alone in the present study (19.4% in men and 9.0% in women; Table S1) seemed to be lower than among those who live alone on remote islands of Nagasaki (40.5% in men and 29.4% in women),^15^ although the questionnaires were different. Therefore, the physical activity in our participants might have contributed to the reduced frailty prevalence in the present study. Nevertheless, as evidence concerning residents of remote islands is scarce and our sample size was small, further investigations are needed.

In this study, we found that living arrangements are independently associated with not only physical frailty but also prefrailty, after adjusting for potential confounding factors. Furthermore, we showed stepwise deterioration of objectively measured physical functions from living with a spouse/partner to living alone. These findings suggest that in such remote islands, living with spouse might be desirable to prevent (pre)frailty. We speculate that these results might be related to cultural/traditional features in Japan. First, marriage is traditionally understood that can bring health benefits through providing emotional intimacy, economic benefits, social control of behavior, and more social integration,^33^ which will in turn influence the health outcome of the older people. Perhaps this association might be stronger in rural areas (especially in remote island, which is geographically isolated) than in urban areas. Second, the fewer choices in housing units, such as nursing homes that can accommodate single living older adults or visiting nurse stations in rural areas, might be related to these results. In Japanese culture, most older people tend to choose to live with their family members when they become single. In this sense, living conditions among those who live alone may have difficulties to be accommodated or supported by communities; therefore, older people living alone might become physically poorer than counterparts on remote islands. Interestingly, while living alone in our sample (20.0%) is similar to that in previous remote island study (22.9%),^15^ this number is slightly higher than other rural, but not remote, island studies in Japan (16.3%^34^ and 15.8%^35^). It is difficult to discuss how these greater numbers of living alone on remote islands were associated with physical frailty, but it is clear that further investigations are needed to explain how these living arrangements and/or health disadvantages in rural, especially on remote, islands are associated with frailty, to conclude our findings.

In the present study, gender‐stratified analyses (Table 4) showed that both living alone and living with other family members (not with a spouse) were associated with a risk of frailty in older men, but not in women, even after adjusting for covariates. The fact that living alone is only associated with frailty among men is consistent with the results of a recent meta‐analysis.^16^ We speculate that these gender‐specific results may be associated with characteristics of the Japanese lifestyle, such as cooking, shopping, and housework. In Japan, women have traditionally performed most of the housework; thus, Japanese men tend to be unfamiliar with cooking. Men who live alone therefore might eat less than men who live with others. A previous study showed that older men who ate alone were more likely to be underweight than men who ate with others.^36^ Based on these findings, living without a spouse might be associated with frailty. However, our male sample was quite small, so the influence of living alone might have been underestimated.

The strengths of our study include extensive investigation of physical functions, such as gait performance, using validated computerized assessment tools, together with the assessment of physical frailty components. The physical function assessments used in the present study (gait speed and grip strength) are clinically convenient and can produce strong evidence of many health impairments.^37^ Additionally, we assessed gait speed using a sheet‐type sensor. Usually, gait speed is measured using a stopwatch, which may result in measurement errors. Thus, the gait speed data obtained in the present study are quite reliable.

The present study has some limitations. The main limitation is the study’s cross‐sectional design, which limits causal inferences. In the future, longitudinal studies that investigate the effects of living arrangements and that employ a prospective design are warranted. Second, we could not compare rural (remote island) and urban areas. Therefore, the findings should be interpreted carefully. To generalize these findings to other areas, further data are needed. The study participants were not randomly sampled from the town. Our study participants were limited to those who participated in the annual health examinations; thus, they might have been more conscious of their health than the general older adult population. This might explain why the slightly lower prevalence of frailty was observed in this study, despite having poor access to healthcare services and a slightly higher rate of living alone than in other rural areas. Nevertheless, the usual gait speed of individuals in rural areas is similar to that of individuals in urban areas.^38^ At least physical functions among our participants seemed to be like general older people. However, the objective gait speed as measured in the present study has not yet been fully validated; hence, the usual gait speed measured using the sheet sensor described in this study may therefore include potentially rough estimates of these relationships. Third, a previous meta‐analysis found that a lack of a social network was associated with physical frailty.^16^ Analyzing the lack of a social network among older adults may help explain the mechanisms that influence physical functions. Finally, because of its convenience, we used the modified version of the frailty phenotype with J‐CHS criteria to assess frailty in Japanese individuals. Because a previous review reported that a modification of the frailty phenotype affects the classification and predictive ability,^39^ the outcome in the present study might need to be interpreted carefully.

## 5 CONCLUSIONS

The prevalence of frailty was 6.6% in older adults living in a remote island in rural Japan. It is notable that the number is comparable to that in urban areas. Furthermore, living alone may correlate with increased risks of frailty and prefrailty. As we observed a greater number of people living alone on remote islands than other rural area (not islands), it seems that older people living with spouse might be desirable to prevent (pre)frailty in these islands. Clearly, further studies are needed to generalize our findings, such as urban–rural comparisons, and a deeper investigation of versatile form of living arrangements would be needed.

## CONFLICT OF INTEREST

The authors declare no conflict of interest.

## Supporting information


Table S1
