# Supplementary material for: Associations between physical frailty and living arrangements in Japanese older adults living in a rural remote island: The Shimane CoHRE study
Source: J Gen Fam Med. 2022 Apr 10;23(5):310–8. doi: 10.1002/jgf2.544 (PMC9444019; doi:10.1002/jgf2.544)
Supplement: Supplementary file 1 — Table S1 [file JGF2-23-310-s001.docx]

| Supplement Table 1. Sex-stratified results of a comparison of the body compositions, physical functions and frailty parameters between by living arrangements | | | | | | | | | | | |
| --- | --- | --- | --- | --- | --- | --- | --- | --- | --- | --- | --- |
|  |  |  |  |  |  |  |  |  |  |  |  |
| Men (n = 245) | | | | | | | | | | | |
|  | With whom you live | Spouse/domestic partner (n = 198) | |  | Other family (n = 16) | |  | Alone (n = 31) | | p-value | Pairwise comparisons |
|  |  |  |  |  |  |  |  |  |  |  |  |
|  |  | Mean | SD |  | Mean | SD |  | Mean | SD |  |  |
| Age (years) |  | 75.1 | 6.2 |  | 77.0 | 9.0 |  | 75.5 | 7.2 | 0.509 |  |
| Body mass index (kg/m^2^) | | 23.2 | 2.8 |  | 23.2 | 3.4 |  | 23.8 | 3.4 | 0.607 |  |
| Muscle mass (kg) | | 20.2 | 2.8 |  | 20.0 | 3.8 |  | 20.3 | 3.2 | 0.950 |  |
| Skeletal muscle index (kg/m^2^) | | 7.6 | 0.9 |  | 7.6 | 1.2 |  | 7.8 | 1.1 | 0.567 |  |
| Grip strength (kg) | | 35.2 | 5.8 |  | 32.6 | 7.0 |  | 31.9 | 6.5 | 0.008 | #: SP vs. AL |
| Gait speed (cm/sec) | | 113.6 | 18.0 |  | 109.2 | 18.2 |  | 103.9 | 22.1 | 0.023 | #: SP vs. AL |
| **Frailty** |  | n | % |  | n | % |  | n | % | p-value |  |
| **Frailty** |  |  |  |  |  |  |  |  |  |  |  |
| Robust (n, %) | | 115 | 58.1 |  | 6 | 37.5 |  | 13 | 41.9 | <0.001 |  |
| Prefrailty (n, %) | | 77 | 38.9 |  | 7 | 43.8 |  | 11 | 35.5 |  |  |
| Frailty (n, %) | | 6 | 3.0 |  | 3 | 18.8 |  | 7 | 22.6 |  |  |
| **Frailty Index items** | |  |  |  |  |  |  |  |  |  |  |
| Weight loss |  | 19 | 9.6 |  | 4 | 25.0 |  | 9 | 29.0 | 0.004 |  |
| Exhaustion |  | 24 | 12.1 |  | 3 | 18.8 |  | 6 | 19.4 | 0.446 |  |
| Physical inactivity | | 22 | 11.1 |  | 4 | 25.0 |  | 6 | 19.4 | 0.153 |  |
| Lower grip strength | | 13 | 6.6 |  | 3 | 18.8 |  | 6 | 19.4 | <0.001 |  |
| Lower gait speed | | 42 | 21.2 |  | 4 | 25.0 |  | 10 | 32.3 | 0.387 |  |
| Women (n = 411) | | | | | | | | | | | |
|  | With whom you live | Spouse/domestic partner (n = 242) | |  | Other family (n = 69) | |  | Alone (n = 100) | | p-value | Pairwise comparisons |
|  |  |  |  |  |  |  |  |  |  |  |  |
|  |  | Mean | SD |  | Mean | SD |  | Mean | SD |  |  |
| Age (years) |  | 74.3 | 5.8 |  | 78.8 | 6.6 |  | 77.3 | 6.2 | < 0.001 | #: SP vs. FA, #: SP vs. AL |
| Body mass index (kg/m^2^) | | 23.0 | 3.4 |  | 22.8 | 2.9 |  | 22.7 | 3.1 | 0.701 |  |
| Muscle mass (kg) | | 14.1 | 2.2 |  | 13.2 | 1.5 |  | 13.5 | 1.8 | 0.001 | #: SP vs. FA, #: SP vs. AL |
| Skeletal muscle index (kg/m^2^) | | 6.3 | 1.0 |  | 6.0 | 0.6 |  | 6.0 | 0.7 | 0.010 | #: SP vs. AL |
| Grip strength (kg) | | 22.8 | 4.0 |  | 22.1 | 3.9 |  | 21.6 | 4.7 | 0.050 | #: SP vs. AL |
| Gait speed (cm/sec) | | 114.9 | 22.9 |  | 107.7 | 23.4 |  | 110.2 | 25.2 | 0.045 |  |
| **Frailty** |  | n | % |  | n | % |  | n | % | p-value |  |
| **Frailty** |  |  |  |  |  |  |  |  |  |  |  |
| Robust (n, %) | | 130 | 53.7 |  | 27 | 39.1 |  | 35 | 35.0 | 0.013 |  |
| Prefrailty (n, %) | | 96 | 39.7 |  | 38 | 55.1 |  | 58 | 58.0 |  |  |
| Frailty (n, %) | | 16 | 6.6 |  | 4 | 5.8 |  | 7 | 7.0 |  |  |
| **Frailty Index items** | |  |  |  |  |  |  |  |  |  |  |
| Weight loss |  | 19 | 7.9 |  | 7 | 10.1 |  | 13 | 13.0 | 0.313 |  |
| Exhaustion |  | 46 | 19.0 |  | 14 | 20.3 |  | 16 | 16.0 | 0.74 |  |
| Physical inactivity | | 27 | 11.2 |  | 8 | 11.6 |  | 9 | 9.0 | 0.813 |  |
| Lower grip strength | | 21 | 8.7 |  | 11 | 15.9 |  | 26 | 26.0 | <0.001 |  |
| Lower gait speed | | 65 | 26.9 |  | 22 | 31.9 |  | 31 | 31.0 | 0.606 |  |
| Data mean ± standard deviation (SD). | |  |  |  |  |  |  |  |  |  |  |
| We compared the prevalence by living arrangements using one-way analysis of variance for quantitative data and χ2-test for categorical data. | | | | | | | | | | | |
| SP: Spouse/domestic partner, FA: family, AL: alone. #: p <0.05. | | | |  |  |  |  |  |  |  |  |
